# Beneficial Effect of Metformin on the Five-Year Survival in about 40,000 Patients with Head and Neck Cancer

**DOI:** 10.3390/cancers16050982

**Published:** 2024-02-28

**Authors:** Florian Gaertner, Saskia Preissner, Max Heiland, Robert Preissner, Jonas Wüster

**Affiliations:** 1 Department of Oral and Maxillofacial Surgery, Campus Virchow-Klinikum, Charité—Universitätsmedizin Berlin, Corporate Member of Freie Universität Berlin, Humboldt-Universität zu Berlin and Berlin Institute of Health, Augustenburger Platz 1, 13353 Berlin, Germanyjonas.wuester@charite.de (J.W.); 2 Institute of Physiology and Science-IT, Charité—Universitätsmedizin Berlin, Corporate Member of Freie Universität Berlin, Humboldt-Universität zu Berlin and Berlin Institute of Health, Philippstr. 12, 10115 Berlin, Germany; robert.preissner@charite.de

**Keywords:** metformin, diabetes, HbA1c, head and neck cancer, survival, real-world evidence

## Abstract

**Simple Summary:**

Metformin is well known for its glucose-lowering effect and is mainly prescribed for diabetes type II. Moreover, some studies showed a beneficial effect on the survival of patients with head and neck cancer with metformin medication. Therefore, this study aimed to investigate the effect of metformin medication on five-year survival in such patients using a federated network of more than 80 healthcare organizations. Two cohorts matched by age, gender, BMI, diabetes type 2, and risk factors were built: the patients with head and neck cancer on metformin were assigned to cohort I, and the patients with head and neck cancer not on metformin medication were assigned to cohort II. We found a higher five-year survival rate for cohort I, indicating the beneficial effect of metformin medication on five-year survival. Since this study was conducted retrospectively, further clinical research is required.

**Abstract:**

Introduction: Even in times of new therapy regimes, the overall survival of patients with head and neck cancer remains low. Since the previous studies showed the beneficial effect of metformin medication on the survival of patients with cancer, our objective was to investigate if—and in which way—metformin medication affects the overall survival of patients with head and neck cancer. Methods: Clinical data pertaining to patients diagnosed with head and neck cancer (International Classification of Diseases 10 codes C00-C14, C31, and C32) were retrospectively retrieved from the TriNetX network (TriNetX, Cambridge, MA, USA). The initial cohort extracted from the network was stratified into two groups: patients on metformin medication (cohort I), and individuals not on metformin medication (cohort II). The matching criteria included age, gender, BMI, type 2 diabetes, and risk factors, such as nicotine and alcohol abuse/dependence. Kaplan–Meier analysis, risk analysis, and the calculation of odds and hazard ratios were conducted. Additionally, the Hemoglobin A1c values were subject to analysis. Results: Following matching, each cohort comprised 20,416 patients. Cohort I exhibited a higher five-year survival rate at 75.3%, in contrast to cohort II, which registered a rate of 69.8%. The odds ratio was 0.79 (95% CI = 0.75–0.83), and the hazard ratio was 0.78 (95% CI = 0.75–0.82). Conclusion: Metformin medication may correlate with improved five-year survival rates in patients with head and neck cancer. Since potentially influencing factors such as comorbidities and the initial tumor stage were not available, the results of our retrospectively conducted study must be interpreted with caution.

## 1. Introduction

Among all the epithelial malignancies affecting the oral cavity, pharynx (encompassing naso-, oro-, and hypopharynx), larynx, nasal cavity, paranasal sinus, and salivary glands, head and neck cancer (HNC) ranks as the seventh most-prevalent tumor type globally [1]. Within the heterogenous group of HNC, almost 90% represents head and neck squamous cell carcinoma (HNSCC), deriving from the mucosal epithelium of the upper aerodigestive tract [2]. The traditional risk factors, such as heavy tobacco and/or alcohol consumption, are known to be related to HNC [3] and are most likely associated with the majority of all HNCs (~75%) [4]. Lately, the incidence of human papillomavirus (HPV)-associated HNC has increased, especially in younger people diagnosed with oropharyngeal squamous cell cancer (OPSCC) [5]. Owing to advancements in radiotherapy, the incorporation of concurrent radio-sensitizing systemic therapy, and the application of definitive radiotherapy or chemoradiotherapy, there has been an augmentation in the overall survival (OS) rates for OPSCC [3,5]. Nevertheless, a high level of disease recurrence, especially in HPV-negative and/or smoking patients, occurs, reflecting the need for new chemo-preventive treatment modalities to improve the survival of such patients [6,7].

Metformin (*N*, *N*-dimethyl biguanide) belongs to the biguanide class of anti-diabetic drugs and has been used since 1957 for the treatment of hyperglycemia in Europe and since 1994 in the U.S. [8]. Due to its glucose-lowering effects and its superior safety profile, metformin has become a first-line therapy for the treatment of type II diabetes [9] and is the most widely prescribed anti-diabetic medication worldwide [10]. The major glucose-lowering effect is mainly mediated through the inhibition of hepatic gluconeogenesis, while the underlying mechanism is still not fully understood [8,9].

The influence of metformin on diseases other than diabetes, such as cancer, are of particular interest in current scientific studies. Other recent prospective studies show contradictory results with no improvement in the outcome of patients with metformin medication [11,12,13]. These findings refer to patients suffering from unresectable stage III non-small-cell lung cancer (NSCLC) [11], patients with high-risk nonmetastatic breast cancer receiving standard therapy [12], and patients with advanced pancreatic cancer treated with gemcitabine and erlotinib [13]. Moreover, metformin use in patients with locally advanced NSCLC treated with chemotherapy even worsened the outcome and led to an increased number of toxic effects [14]. Nevertheless, the protective effect of metformin has been described in various in vivo and in vitro studies, indicating a direct effect on cancer cells and an indirect effect on the host, resulting in anti-cancer activity [10,14,15]. Regarding the direct effect of metformin, Dowling et al. examined the influence of metformin on breast cancer cells and showed the inhibition of the AMPK/mTOR pathway and a subsequent reduction in translation initiation [15]. For HNSCC cell lines, other recent studies not only revealed the cytotoxicity of metformin, but also an associated reduction in cell viability (under 50%) [16,17]. Further, some in vitro studies indicate that metformin provokes persistence in the G0/G1 phase (cell cycle regulation) of HNSCC cells, resulting in apoptosis [16,18]. Additionally, the indirect effect of metformin use on cancer is most likely achieved by the associated lower blood glucose level and the anti-inflammatory/immunological effect [10,14,19,20].

Regarding other malignities than HNC, the previous studies revealed, e.g., a reduced cancer-specific mortality among women with breast cancer, colorectal cancer, or endometrial cancer [21]. Some analyses even suggest that metformin might be a useful adjuvant agent, particularly in colorectal and prostate cancers [10]. Concerning HNC, a systematic review and meta-analysis by Jiao et al. showed a significant improvement in the OS of patients with metformin medication, thus suggesting metformin as an adjunct to the treatment of HNC [22]. Jiao et al. included 11 studies with 14,694 participants [22], which might result in a heterogenous study collective. Here, the subgroup analysis of age indicated a benefit for patients younger than 65 years. Further subgroup analysis of the comorbidities showed that metformin medication was only linked to significantly improved patients’ outcomes for studies without an adjustment for comorbidities [22]. Therefore, Jiao et al. emphasized the need for future studies with a larger sample size [22].

Therefore, using the TriNetX Global Health Research Network (TriNetX, Cambridge, MA, USA), a so-called real-world database, this study aims to investigate the influence of metformin use in a large cohort of almost 40,000 patients with HNC on five-year survival.

## 2. Materials and Methods

### 2.1. Ethics Statement

No ethical review/approval and no written informed consent were required following national legislation and institutional requirements.

### 2.2. Data Acquisition, Inclusion and Exclusion Criteria, and Patient Matching

The TriNetX database houses medical records of over 80 healthcare organizations (HCOs) across 30 countries, primarily used to record and analyze clinical data for research purposes. In this study, data of patients diagnosed with HNC (International Classification of Diseases [ICD]-10 codes C00-C14, C31, and C32) between 5 and 20 years before the access date (18 January 2024) were extracted from the TriNetX Global Health Research Network. This utilized workflow has previously been implemented in our group’s other studies [23,24]. The inclusion criteria required medical records covering at least 5 years (1825 days) of follow-up after visiting the HCO for inpatient encounters. Cohort I and cohort II were formed as follows: cohort I comprised individuals with diagnosed HNC on metformin medication, while cohort II included individuals with HNC not on metformin medication. Subsequently, randomization was achieved by one-to-one matching for age, sex, BMI, nicotine dependence, alcohol abuse/dependence, and diabetes type 2 (ICD-10: Z87.891 and F10.1, or F10.2 and E11).

### 2.3. Data Analysis

This study aimed to evaluate the effect of metformin medication on the survival of patients with HNC. Therefore “death” was defined as the primary outcome.

Statistical methodologies, including Kaplan–Meier survival analysis, Cox proportional hazards regression, risk ratios (RRs), odds ratios (ORs), and hazard ratios (HRs), were employed individually for each cohort. The criteria for patient recovery were defined as the absence or non-recurrence of head and neck cancer (HNC) or metastases within a five-year timeframe. Consequently, the scope of data analysis was restricted to a five-year follow-up period. Statistical analysis utilized the Log-Rank test with the threshold for statistical significance set at 5% (*p* = 0.05). Further, Hemoglobin (HbA1c) levels were measured, with subsequent comparison between cohort I and cohort II.

## 3. Results

### 3.1. Assessment, Allocation, and Matching

**Figure 1:**
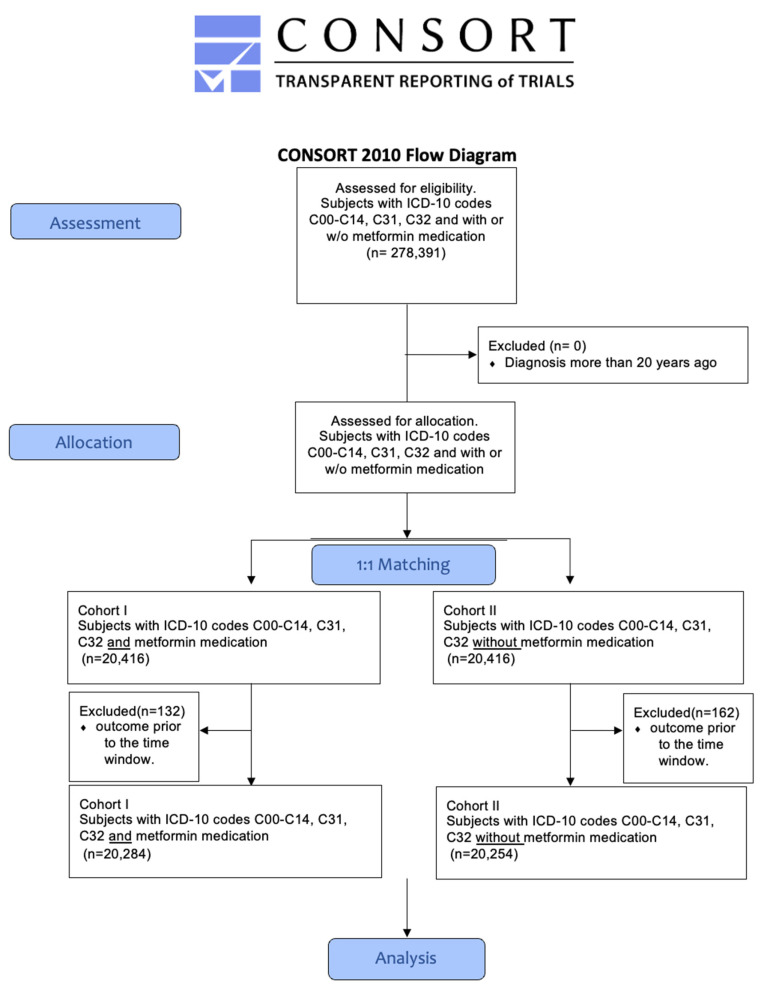
Modified CONSORT flowchart.

In total, 75 HCOs responded, whereby 278,391 patients met the inclusion criteria (ICD-10 codes C00-C14, C31, and C32) and could be retrieved from the database. To eliminate the confounders, a 1:1 propensity score matching for age, sex, BMI, nicotine dependence (ICD-10 code Z87.891), alcohol abuse (F10.1), and alcohol dependence (F10.2), as well as diabetes type 2 (E11) was used. After propensity score matching, 20,416 patients were assigned to cohorts I and II. Of all the included patients, 132 patients in cohort 1 and 162 patients in cohort 2 had to be excluded from the results because they had an outcome before the time window (Figure 1). The patients’ characteristics of both cohorts before and after matching are listed in Table 1.

### 3.2. Patient Survival

**Figure 2:**
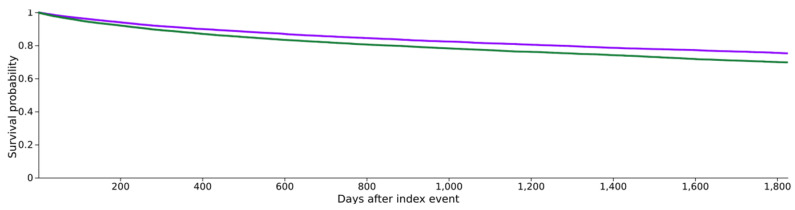
Kaplan–Meier survival curves for both cohorts, with purple indicating cohort I (HNC with metformin medication) and green indicating cohort II (HNC without metformin medication).

**Figure 3:**
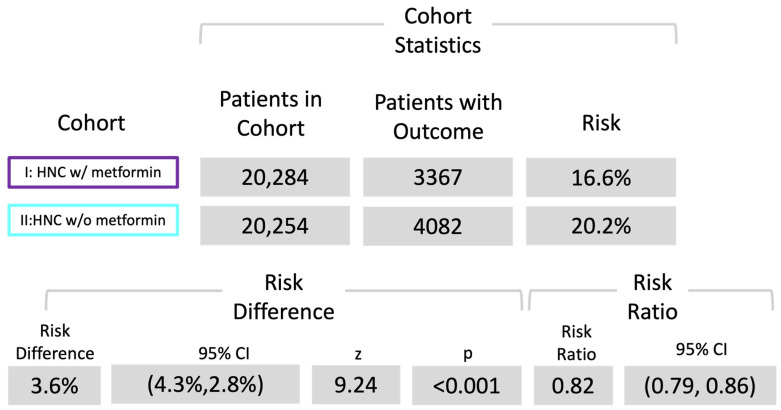
Risk analysis of death. Risk ratio of both cohorts; cohort I (ICD codes C00-C14, C31, and C32 with metformin use) and cohort II (ICD codes C00-C14, C31, and C32 without metformin use).

Following the diagnosis of head and neck cancer (HNC), 3367 patients in cohort I and 4082 patients in cohort II succumbed during the 5-year observation period, corresponding to death rates of 16.6% (cohort I) and 20.2% (cohort II) (see Figure 2). Cohort I demonstrated a superior survival probability at the conclusion of the 5-year timeframe, registering 75.3%, compared to cohort II, which exhibited a survival rate of 69.8% (see Figure 2). The associated risk ratio (RR) was 0.82 (95% confidence interval (CI): 0.79–0.86), while the odds ratio (OR) and hazard ratio (HR) were 0.79 (95% CI: 0.75–0.83) and 0.78 (95% CI: 0.75–0.82), respectively (see Figure 3).

### 3.3. HbA1c

Most recent HbA1c values in the time window were retrieved from 15,305 patients. Here, we found slight differences between both the groups, with a higher mean value (±standard deviation) of 6.96% ± 1.6 in the metformin group when compared to the following value for the group without metformin: 6.42% ± 1.57 (*p* < 0.001).

## 4. Discussion

This investigation explores the association between metformin medication and the five-year survival rate in patients with HNC. To date, contradictory data exist on this specific topic; therefore, this study was conducted to address this question for larger cohorts. Our data indicate a beneficial effect regarding the survival probability five years after initial diagnosis for diabetic patients with HNC on metformin medication compared to the patients with HNC not on metformin medication.

Metformin medication and their effect on malignant tumor diseases have been repeatedly and controversially discussed in the literature. In this context, randomized clinical trials about metformin use in nondiabetic patients with cancer exist, whereby the patients underwent standard therapy with or without the addition of metformin medication [11,12,13,25]. Due to their study design, these studies certainly provide evidence that metformin alone has most likely a rather low-level impact on the outcome of nondiabetic patients with cancer [11,12,13,25]. To the best of the authors’ knowledge, only a first phase 1 trial combining metformin with chemoradiotherapy in patients with locally advanced HNC exists, showing encouraging rates of OS and progression-free survival (PFS) [26]. Nevertheless, this study is limited by its low number of included patients and limited follow-up period.

For the last decade, metformin use has been repeatedly reported to decrease the risk of HNC development [27,28]. Consequently, growing interest in metformin arose due to its assumed beneficial influence on cancer survival outcomes, which has already been evaluated in previous studies about other cancer types [10,21]. In this regard, most studies revealed higher survival rates for patients using metformin, e.g., for pancreatic, prostate, and gynecologic malignities [10,21]. Moreover, the other studies even revealed a beneficial influence of post-diagnosis metformin use, e.g., on survival and progression-free survival in lung cancer [29], as well as a survival benefit for patients with pancreatic cancer when metformin was used in an adjuvant setting [30]. Lately, Jiao et al. showed that metformin might be relevant for patients with HNC as an adjuvant treatment since (post-diagnosis) metformin improved the prognosis of patients with HNC [22]. Nevertheless, clinical data on metformin use and its impact on HNC/HNC survival are still the subject of controversy in the literature [22,31]. Our retrospective case–control study concentrated on patients with HNC who continued to take metformin pre-diagnosis, during the post-diagnostic stage, and until the end of the observation period (5 years); this was equivalent to most other studies about the impact of metformin use on the survival of patients with cancer [32,33]. In about two-thirds of cohort I, metformin was prescribed to patients with diabetes type II. The other common indications for metformin use are prediabetes, obesity, and polycystic ovary syndrome. Our cohorts were not adjusted for other comorbidities. Comorbidities play an important role in survival [22] and should therefore be included in future research.

For the patients with diabetes suffering from HNC and on metformin medication, our study provides clinical data, indicating a higher five-year survival rate when compared to that of the patients with HNC without metformin medication. Moreover, due to the well-known influence of glucose metabolism and of the glucose blood-sugar level [34], we analyzed the distributions of HbA1c values in both the cohorts. There are suggestions that reduced blood glucose levels may reduce tumor growth. Since the HbA1c values for patients using metformin were higher when compared to the group without metformin medication (6.92% ± 1.53 vs. (6.38% ± 1.47); (*p* < 0.001)), the metformin-based reduction of blood sugar alone as a mechanism of tumor suppression might not be reasonable. In addition to other (possibly unknown) targets, the direct effect on cancer cells is most likely carried out by the inhibition of the AMPK/mTOR pathway [15], and the indirect effect is caused by a combination of the blood glucose-lowering properties, as well as immunological and anti-inflammatory effects [10,14,19,20].

Given the retrospective initiation of this study, the TriNetX database was utilized to identify subjects with head and neck cancer (HNC) diagnoses, defined by ICD-10 codes C00-C14, C31, and C32 [23,24]. The accurate classification of malignant neoplasia was assumed in this context. Furthermore, the absence of data pertaining to initial staging, in accordance with the Union for International Cancer Control (UICC), was noted, despite its potential impact on the observed survival rates. As a result, the applied therapy, as well as clinical, histological, and molecular features, could not be considered [35]. Therefore, the results of this study must be cautiously interpreted as these factors represent clear limitations. Additionally, since the data were derived from various HCOs, details on tobacco use (total pack-years) and alcohol abuse (consumed alcohol units) may be inconsistent, which might cause a certain risk of confounder bias, even though one-to-one matching was performed. It has to be mentioned that matching for age resulted in a 0.5-year-older cohort II and the matching for sex resulted in 1.7% more females in cohort II. As younger age and the female gender are positive prognostic factors for survival, the trend of our results may be enhanced. Matching for BMI resulted in an elevated BMI of 30.7 for cohort I compared to 28.2 for cohort II. A higher BMI may also be regarded as a positive prognostic factor in patients with HNC.

The limitations inherent in this study were addressed through the inclusion of substantial cohorts, each comprising 20,416 patients, and the meticulous one-to-one matching approach aimed at mitigating the potential differences. Additionally, the data retrieved from the TriNetX database adhere to the rigorous standards of the National COVID Cohort Collaborative N3C, thereby attesting to the high quality of the obtained data.

The other studies on this topic included considerably fewer patients [22,31,36,37,38]; in contrast, this study provides clinical data on about 40,000 patients.

Consequently, the favorable impact observed in our study regarding the pre-diagnostic use of metformin on the survival of patients with head and neck cancer (HNC) may provide novel perspectives on this contentious subject, potentially motivating additional investigations in this area. There is a clear need for advanced research, especially of prospective randomized controlled trials since the comparable studies showed no advantage of metformin use for different types of cancer, such as unresectable stage III NSCLC [11], high-risk nonmetastatic breast cancer [12], or advanced pancreatic cancer [13]. Therefore, prospective randomized controlled trials are highly needed.

## 5. Conclusions

Among the patients with HNC, the patients with diabetes receiving metformin medication had an elevated five-year survival rate. These findings need to be confirmed in future studies. Future investigations should include an adjustment for comorbidities.

## Figures and Tables

**Table 1:** Attributes of cohort I and cohort II, both prior to and following matching for variables, such as age, gender, BMI, tobacco use, and alcohol abuse. * One hundred and thirty-two patients in cohort I had to be excluded from the results because they had an outcome before the time window; ** one hundred and sixty-two patients in cohort II patients had to be excluded from the results because they had an outcome before the time window.

	Before Matching				After Matching			
Patients (*n*)	Cohort I	Cohort II	*p*-Value	Standardized Mean Difference	Cohort I *	Cohort II **	*p*-Value	Standardized Mean Difference
Total	22,028	256,363	<0.001	0.280	20,416	20,416		
Female	6774 (32.6%)	70,923 (32.7%)	0.063	0.013	6264 (30.7%)	6617 (32.4%)	<0.001	0.04
Male	14,577 (66.6%)	158,055 (67.7%)	0.002	0.022	13,653 (66.9%)	13,316 (65.2%)	0.04	0.02
Mean age at diagnosis (years)	65.0 ± 11.5	61.2 ± 15.6	<0.001	0.28	65 ± 11.6	65.5 ± 12.6	<0.001	0.04
BMI	30.7 ± 6.7	26.9 ± 6.1	<0.001	0.59	30.7 ± 6.7	28.2 ± 6.6	<0.001	0.36
Diabetes II	13,932 (63.7%)	13,606 (5.8%)	<0.001	1.53	12,471 (61.1%)	12,471 (61.1%)	1.0	<0.001
Nicotine dependence	4812 (22.0%)	15,625 (6.7%)	<0.001	0.45	4357 (21.3%)	4217 (20.7%)	0.09	0.02
Alcohol abuse	978 (3.5%)	6153 (2.6%)	<0.001	0.09	919 (4.5%)	957 (4.7%)	0.3	0.01
Alcohol dependence	766 (3.6%)	5058 (2.2%)	<0.001	0.08	764 (3.7%)	824 (3.9%)	0.13	0.02

## Data Availability

Data are contained within the article.
